# Spinal Injections: A Narrative Review from a Surgeon’s Perspective

**DOI:** 10.3390/healthcare11162355

**Published:** 2023-08-21

**Authors:** Dong Ah Shin, Yoo Jin Choo, Min Cheol Chang

**Affiliations:** 1 Department of Neurosurgery, Spine and Spinal Cord Institute, Severance Hospital, Yonsei University College of Medicine, 50-1, Yonsei-ro, Seodaemun-gu, Seoul 03722, Republic of Korea; cistern@yuhs.ac; 2 Department of Physical Medicine and Rehabilitation, College of Medicine, Yeungnam University, Nam-gu, Daegu 42415, Republic of Korea; cyj361@hanmail.net

**Keywords:** spinal pain, injection, surgery, spine, conservative treatment

## Abstract

Spinal pain is one of most frequent complaints of the general population, which can cause decreased activities of daily living and absence from work. Among numerous therapeutic methods, spinal injection is one of the most effective treatments for spinal pain and is currently widely applied in the clinical field. In this review, spinal injection is discussed from a surgeon’s perspective. Recently, although the number of spinal surgeries has been increasing, questions are arising as to whether they are necessary. The failure rate after spinal surgery is high, and its long-term outcome was reported to be similar to spinal injection. Thus, spinal surgeries should be performed conservatively. Spinal injection is largely divided into diagnostic and therapeutic blocks. Using diagnostic blocks, such as the diagnostic selective nerve root block, disc stimulation test, and diagnostic medial branch block (MBB), the precise location causing the pain can be confirmed. For therapeutic blocks, transforaminal nerve root injection, therapeutic MBB, and percutaneous epidural neuroplasty are used. When unbearable spinal pain persists despite therapeutic spinal injections, spinal surgeries can be considered. Spinal injection is usefully used to identify the precise location prior to a patient undergoing injection treatment or surgery and can reduce pain and improve quality of life, and help to avoid spinal surgery. Pain physicians should treat patients with spinal pain by properly utilizing spinal injection.

## 1. Introduction

Spinal pain is highly prevalent, often resulting in many lost work days. Degenerative changes in the spine begin to occur in adults in their twenties, starting with intervertebral disc degeneration and extending to end plates, vertebrae, and facet joints located in the posterior spinal region [1]. When these degenerative changes affect the central spinal canal or the intervertebral foramen, central spinal canal stenosis and foraminal stenosis develop [2,3]. The pain resulting from spinal degeneration reduces quality of life and, in severe cases, can cause depression [4]. In cases of severe spinal pain, caregivers experience a significant burden in providing care to patients [5,6]. Therefore, to treat pain of spinal origin, many clinicians and researchers aim to identify the mechanism of pain generation to provide targeted and more effective treatment. Various spinal surgical techniques have been developed, in addition to conservative methods, to control pain of spinal origin [7,8]. Among the conservative treatment methods, spinal injection is one of the most effective treatments for pain and is currently widely applied in the clinical field. In this review, spinal injection is discussed from a surgeon’s perspective.

## 2. Increase in Spinal Surgeries

In 1934, Mixter and Barr published a case study concerning a 28-year-old patient with sciatica, a positive straight leg raise test, and an absent ankle reflex, who they preoperatively diagnosed with a herniated lumbar disc (HLD). His pain was treated through removal of a 1 cm ruptured disc with laminectomy [9]. This was the first case study to report a preoperative provisional diagnosis of HLD. Since then, spinal surgery techniques have developed globally and the number of spinal surgeries has rapidly increased. According to recent policy trends reported by the Health Insurance Review and Assessment Service of Korea, there was a 47% increase in the number of spinal surgeries from 2007 to 2013 [10]. In the United States, the number of elective lumbar fusions has increased by 62.3% (32.1% per 100,000 US adults), with 122,679 cases (60.4 per 100,000) reported in 2004 and 199,140 cases (79.8 per 100,000) reported in 2015 [11].

This recent surge in the number of spinal surgeries performed may be explained as follows. First, the availability of magnetic resonance imaging (MRI) has led to a larger number of findings concerning spinal abnormalities. However, very few older adults are likely to be without some form of degenerative change or pathology in their spines [12]. Some studies have reported that abnormalities were observed on lumbar MRI in two-thirds of asymptomatic people, with HLDs observed in 76% of those without back pain, whereas 13% were found to have disc rupture [11,12]. Moreover, spontaneous regression of herniated disc tissue can occur, and can completely resolve after conservative treatment. The rate of complete resolution of HLD was reported be to 43% for sequestrated discs and 15% for extruded discs [13]. Therefore, performing surgery based on MRI findings alone could lead to unnecessary surgeries. Second, the number of hospitals specializing in the treatment of spinal disorders has increased. As of March 2023, 16 Korean hospitals specialize in spinal surgery. As the number of specialists in the field of spinal surgery increases, the number of surgical cases is also likely to increase. Medicine is a specialized field where a demand can potentially be created by medical supply. An increase in the number of doctors per capita has been shown to increase medical costs per capita [14]. Furthermore, an increase in the number of doctors treating spinal diseases is likely to increase the number of spinal surgeries. Third, an increase in the number of spinal surgeries is related to profit motives. Spinal surgery has been reported to be a field involving substantial profit [15]. Fourth, due to the aging population, the number of patients with spinal diseases has increased. Living longer has led to an accumulation of degenerative knee, hip, and spinal changes that manifest as diseases [16]. Fifth, the scope and time spent in activities in modern populations have increased. Humans are awake for longer, leading to increased activity time; thus, exposing the spine to the forces of gravity for more extended periods [17]. In particular, it has been revealed that sedentary lifestyle and decreased activity in modern society are associated with degenerative spinal diseases [18,19]. This has also led to an increased incidence of spinal diseases and an increase in the number of spinal surgery cases [20,21].

## 3. Questions Arising Concerning the Necessity of Spinal Surgery

If spinal surgery can fully resolve spinal pain, then an increase in the number of spinal surgeries performed need be of no concern. However, not all spinal surgery outcomes are positive and many patients who have undergone spinal surgery experience symptomatic complications [22,23,24]. Voelker et al. evaluated the complications after 282 spinal injections [25]. A total of 131 minor treatment-related events, including transient pain at the injection site, radiating pain, and nerve root irritation, occurred. However, no persistent neurologic deficits were reported. Furthermore, in one study, in which patients were asked whether they would consider undergoing another spinal surgical intervention, five times as many people who had undergone multiple spinal surgeries responded negatively compared with those who had undergone a limited number of spinal surgeries [26]. In addition, Deyo et al. reported that the number of spinal surgeries performed was five times higher in the United States than that in the United Kingdom [27], despite there being no studies reporting that people in the United Kingdom experience more spinal pain than those living in the United States. One study reported that patients who had undergone nucleotomy were three times more likely to receive a subsequent fusion than those who had not [28]. This finding suggests that an increased number of surgical interventions has led to more patients experiencing spinal diseases or complications. Minimally invasive spine surgery, which minimizes the scope of spinal surgery, has recently been receiving attention; however, it too is not without challenges [7,8]. Minimally invasive spine surgery is less likely to cause perioperative morbidity as there is minimal tissue damage; however, success rates have been reported to be similar to those of open surgery [29,30]. Moreover, in Alvi’s systematic review, tubular discectomy was found to have a greater rate of overall complication (odds ratio: 1.49), greater incidence of dural tears (odds ratio: 1.72), and recurrent herniation (odds ratio: 2.09), compared with open surgery [31]. Furthermore, Zhao et al. compared percutaneous transforaminal endoscopic discectomy with microendoscopic discectomy, which is closer to microscopic discectomy [32]. They reported that percutaneous transforaminal endoscopic discectomy had a higher recurrence rate (odds ratio: 1.60).

## 4. Spinal Surgery Outcomes

Many patients with spinal stenosis, HLD, or discogenic back pain have persistent pain even after surgical operation [33,34,35]. Alhaug et al. reported the outcome of spinal surgery in 8258 patients with spinal stenosis [33]. At a 12-month follow-up, the outcomes of 20% of the patients were classified as failures and the symptoms of 6% of the patients were worse. Parker et al. evaluated the therapeutic outcome after discectomy for HLD using a systematic review [35]. They included 21,180 patients with HLD in 90 studies, reporting a 5–36% recurrence rate of back or leg pain 2 years after discectomy. Furthermore, Mirza et al. recruited 86 patients who had surgery (instrumented fusion, disc replacement, laminectomy, or discectomy) for discogenic back pain [34]. They reported that the 1-year success rate was 33% and the rate of reoperation was 11%.

In patients with spinal stenosis or a herniated disc, several studies have reported that short-term results of spinal surgery are superior to conservative treatment, but that long-term follow-up results are unsatisfactory [36,37]. One 10-year follow-up study reported that, in terms of efficacy, spinal surgery was not superior to conservative treatment [38]. Furthermore, many patients complain of persistent pain even after surgery. Inoue et al. reported that the prevalence of lower back pain, dull ache, numbness, cold sensations, and paresthesia after spine surgery was 94.0%, 71.1%, 69.8%, 43.3%, and 35.3%, respectively [39]. Parker et al. reported a 5–36% recurrence rate of pain 2 years after discectomy for HLD [35]. Skolasky et al. reported that 29.2% of patients had the same or increased pain 1 year after surgical laminectomy for lumbar stenosis [40].

Failure rates in spinal surgery have been shown to result when operating on a wrong lesion due to an incorrect assessment of the mechanism of pain, leading to a poorer prognosis if complications occur compared with preoperatively, in addition to being ineffective for patients who are not indicated for surgery [41]. In terms of spinal fusion, which is commonly performed for back pain, the failure rate is reported to range between 20% and 40% [39], suggesting that unnecessary or ineffective surgeries need to be actively avoided.

## 5. Efficacy of Injection Treatments

In terms of conservative treatments for spinal diseases, spinal injections, which are widely used in the clinical setting, have been reported to be effective in controlling spinal-disease-related pain [42,43,44]. Injection treatments applied to spinal nerves, excluding those applied to muscle or ligamentous tissues, are discussed here, as most studies have focused on nerve blocks, which have comprised approximately 75% of spine-related injection treatments [45].

The nerve block, first reported by Bogduk [46], involves administering local anesthesia to inhibit the generation or propagation of pain through acting on nerves that signal pain [47]. Steroids contained in the injectate soothe the inflamed nerves and tissues [42]. However, the therapeutic effects of this form of conservative treatment have been challenged given that the herniated disc is not removed nor is the narrowed nerve canal expanded. However, in degenerative spinal disease, good natural outcomes have been reported. While the herniated intervertebral disc is not surgically removed, it is absorbed and decreases in size or disappears and, even if the neural canal is narrow, the nerve adapts to the narrowed spinal canal or spinal foramen [48]. Thus, a patient can be relieved of pain without surgery as long as the acute severe pain phase has resolved. In many cases, pain does not recur once it has resolved. Furthermore, injection treatments can be used for the treatment of residual or unresolved pain that persist after spinal surgery [49].

## 6. Spinal Injection Treatment

The two categories of spinal injection treatment, namely, diagnostic blocks and therapeutic blocks [50,51], are described below.

### 6.1. Diagnostic Blocks

Diagnostic blocks have been used for many years for diagnosis and to facilitate predicting prognosis following spinal injection treatment [51,52].

#### 6.1.1. Diagnostic Selective Nerve Root Blocks

**Figure 1:**
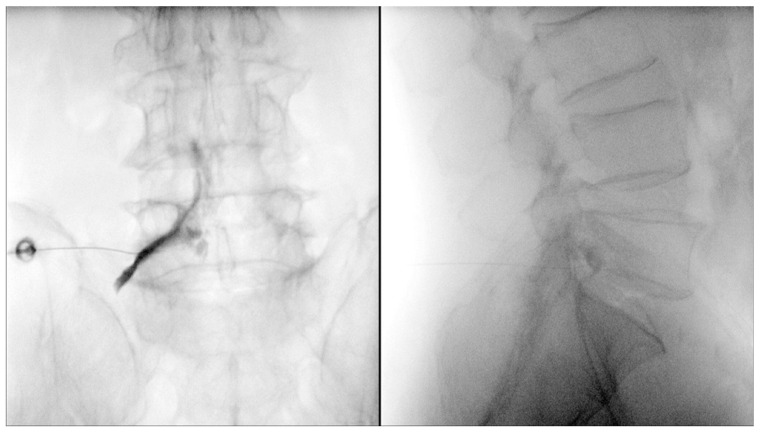
Fluoroscopy-guided lumbar diagnostic selective nerve root blocks (or transforaminal nerve root injection) of Rt. L5 nerve root (Lt.: anteroposterior view, Rt.: lateral view).

Diagnostic selective nerve root blocks can be used to accurately identify which level of intervertebral disc herniation or foraminal stenosis among several spinal levels is the cause of pain (Figure 1) [53]. The cause of pain can be identified through administering a selective diagnostic nerve root block to the lesion suspected as being the cause of pain, then determining whether the pain has disappeared. Diagnostic selective nerve root blocks can be used to identify the nerve root causing radicular pain, with a diagnostic accuracy of 68–91.8% [54]. Diagnostic selective nerve root blocks can be useful when the findings for preoperative imaging studies and clinical presentations are inconsistent, or when there are symptoms without particular findings on images [54]. When a diagnostic selective nerve root block is performed to identify an area that is the cause of pain, it is important to use a low volume injectate to increase the specificity of the diagnosis (recommended local anesthetic volume, 0.5 cc (without steroids)) [55]. It has been reported that the accuracy can be improved if the analgesic effect is simultaneously tested while determining whether a patient’s usual pain is evoked during the diagnostic selective nerve root block [56]. Sasso et al. reported that 90% of patients with positive responses had had a diagnostic selective nerve root block administered prior to cervical and lumbar spine surgery, whereas only 60% of patients with negative responses showed a good outcome [57].

#### 6.1.2. Disc Simulation Tests

**Figure 2:**
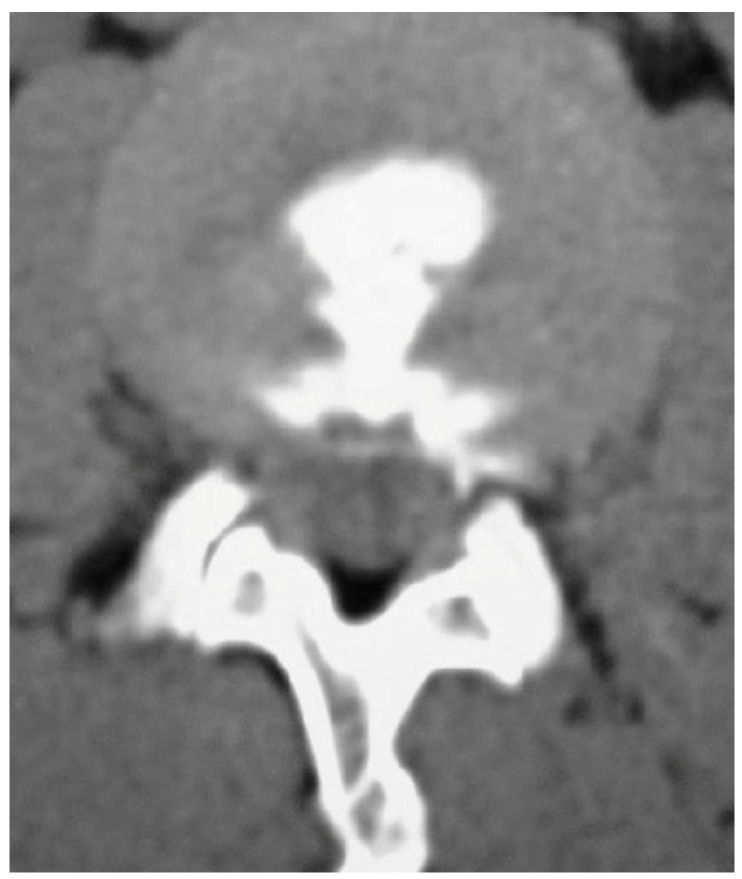
Lumbar provocation discography at L4-5.

When discogenic back pain is suspected, a disc stimulation test (discography) can be used to make a confirmed diagnosis (Figure 2) [58,59]. It is possible to determine whether a disc is the cause of pain through injecting a contrast agent into the disc to increase internal disc pressure and determine whether the same pain is elicited. If the outcome is positive, discogenic back pain can be treated with an intradiscal injection, total disc replacement, or spinal fusion [60,61,62]. Furthermore, a disc stimulation test can identify the pain-causing segment in multiple disc degeneration, can distinguish between recurrent disc herniation and postoperative scarring, and can be performed to identify the causative lesion prior to surgery in far lateral HLDs [59,63,64]. Colhoun et al. reported that the success rate for patients with a positive outcome after an initial discography was 89%, whereas the success rate for patients with a negative outcome was only 52% [65]. Derby et al. used discography to divide discs into chemically sensitive, mechanically sensitive, and negative or intermediate groups, and reported that patients in the chemically sensitive disc group had an 89% success rate for surgery after inter-body fusion; otherwise, the success rate was <20% [66]. The reported findings in these two studies indicate that discography can predict the success rate of surgery and guide the appropriate method of surgery. However, while discography is the only tool that can correlate symptoms and pathology, its efficacy remains to be confirmed as some studies have claimed that it is not useful in prognosis prediction [67].

#### 6.1.3. Diagnostic Medial Branch Blocks (MBBs)

**Figure 3:**
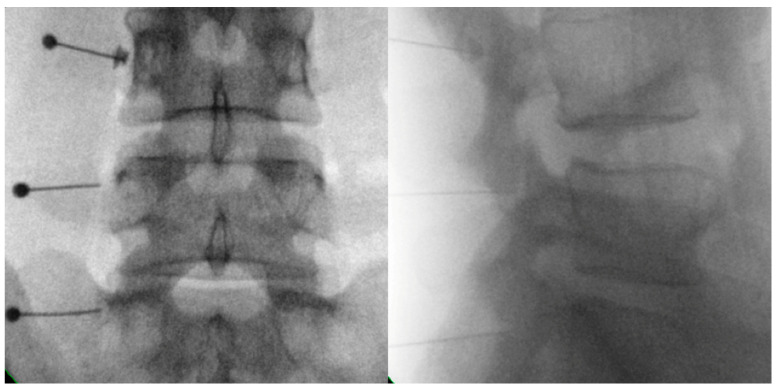
Fluoroscopy-guided diagnostic (or therapeutic) Rt. L3, L4, and L5 medial branch blocks (Lt.: anteroposterior view, Rt.: lateral view).

To diagnose facet-joint-origin pain, a diagnostic MBB is performed (Figure 3) [68]. An intra-articular injection into a facet joint can be performed to help diagnose facet-joint-origin pain; however, a diagnostic MBB is recommended for its diagnosis as the injectate may leak out of the facet joint and, even if injected, the drug may not be completely injected into the facet joint in situations of severe degeneration [69]. Despite this, the MBB has a high false positive rate, which can be resolved through repeating the MBB [70,71,72] using 0.5 cc of lidocaine, bupivacaine, or ropivacaine for injection without mixing steroids, when using MBB for diagnostic purposes [72]. A double comparative block is performed using a short-acting anesthetic such as lidocaine once, followed by a long-acting anesthetic such as bupivacaine or ropivacaine, during which time the patient is requested to complete a one-day pain diary. If there is a meaningful reduction in pain, a diagnosis of pain resulting from facet joint arthritis can, therefore, be made [69]. One facet joint is innervated by three levels of medial branches; one branch each from the two levels above and one branch from the level immediately below. Therefore, for example, medial nerve branches of the 3rd, 4th, and 5th lumbar vertebrae must be blocked for an effective nerve block since lumbar 4 and 5 facet joints are innervated through medial nerve branches from lumbar 3, 4, and 5 nerve roots [73].

### 6.2. Therapeutic Blocks

#### 6.2.1. Transforaminal Nerve Root Injections

Transforaminal nerve root injection can be used to treat radiating pain resulting from a herniated intervertebral disc and radiating pain owing to stenosis-related nerve root compression (Figure 1). A transforaminal nerve root injection typically provides an analgesic effect for two to three months, thus reducing the amount of narcotic analgesics used, and possibly also preventing surgery [74,75]. This treatment can be an alternative to surgery for people who have intervertebral disc herniation or stenosis, but who do not wish to undergo surgery or do not need surgery. In a systematic review by Manchikanti et al., transforaminal nerve root injections showed the best evidence for controlling pain resulting from disc herniation, but only when steroids and local anesthetics were injected in combination [76]. Bhatti et al. reported that >80% of patients with sciatic pain due to LDH could successfully avoid surgery after transforaminal nerve root injection treatment [77]. Kenney et al. conducted a 5-year follow-up after transforaminal nerve root injection treatment [78]. Although transforaminal nerve root injection has a high success rate of 79% at 6 months, the majority of subjects experienced recurrence of symptoms over the next 5 years. However, only a small number of patients required additional injections, surgery, or opioid pain relievers. Furthermore, Wilby et al. reported that transforaminal nerve root injection demonstrated similar effectiveness to microdiscectomy in cases of sciatica persisting for 6 weeks to 12 months [79]. Considering the safety of transforaminal nerve root injection and the cost of surgery, it is recommended to consider transforaminal nerve root injection as a primary option for sciatica without significant neurological deficits within 12 months. They recommended that surgery can be advised for patients who do not respond to transforaminal nerve root injection. However, surgery should be performed if a patient has cleft palate syndrome or foot drop resulting from a herniated disc.

Several studies have reported the efficacy of transforaminal nerve root injections on radiating pain resulting from HLD or spinal stenosis [38,80]. Pulsed radiofrequency and transforaminal nerve root injections can be combined for longer lasting and greater effects when results after performing transforaminal nerve root injection only are unsatisfactory or the effects are short-lasting [81,82].

For transforaminal nerve root injection, the oblique scotty dog subpedicular approach and anteroposterior subpedicular approach can be used [83]. Kaliya-Perumal et al. reported that the oblique scotty dog subpedicular approach took a significantly longer procedural time and a greater number of C-arm exposures [83]. However, the accuracy of needle placement was 95.5% in the oblique scotty dog subpedicular approach and only 72% in the anteroposterior subpedicular approach.

Previous studies related to transforaminal nerve root injections are described in Table 1 [84,85,86,87,88,89,90,91,92,93,94,95,96,97,98,99,100,101].

#### 6.2.2. Therapeutic MBBs

Spinal surgery is effective in relieving radicular pain, but has little effect for axial back pain. If axial back pain is the chief compliant, facet-joint-origin pain can be considered, with the implementation of a therapeutic MBB (Figure 3) [102,103]. This could also reduce pain due to overloading of the facet joint after a compressive vertebral fracture [104]. In clinical practice, some doctors may provide treatment without distinguishing between axial back pain and lumbar radicular pain; however, the different results following surgical treatment for axial back pain and lumbar radicular pain need to be considered. Compared with lumbar radicular pain, for which diagnosis and treatment are relatively clear, axial back pain has various causes and poor treatment outcomes [105]. Therefore, for axial back pain, nonsurgical treatment rather than surgical treatment should first be considered [105]. A common cause of axial back pain is back pain resulting from facet degeneration, and fusion surgery has been considered an excessive treatment in such circumstances [106,107].

Positive therapeutic outcomes after using MBBs for patients with pain originating in a facet joint have been observed in several studies, and MBBs are widely used in actual clinical practice. In double comparative blocks, radiofrequency (RF) neurotomy may be considered if >50% of pain is eliminated in both blocks and if an MBB was performed for treatment purposes with an unsatisfactory outcome and frequent pain recurrence. RF neurotomy has a relatively high success rate in the treatment of facet-joint-origin pain [107,108,109]. If facet-joint-origin pain is clearly diagnosed and the effect of a therapeutic MBB or RF neurotomy is not long-lasting, spinal fusion can be considered.

Studies examining the effectiveness of therapeutic MBBs are described in Table 2 [110,111].

#### 6.2.3. Percutaneous Epidural Neuroplasty (PEN)

PEN can be considered when a transforaminal nerve root injection results in an unsatisfactory treatment effect and a patient declines the offer of surgery. The superiority of PEN has been reported in studies comparing transforaminal nerve root and epidural steroid injections [75,112]. The therapeutic effect of PEN can be sustained for >6 months when administered to appropriate patients [113,114]. PEN is a procedure used to improve symptoms resulting from adhesion detachment and to reduce nerve inflammation via drug administration following catheter insertion into the epidural space through the sacral hiatus [113,114,115]. Mechanisms for reducing back pain and lower extremity radiating pain are known to include neural decompression, washing of inflammatory materials, and effective drug delivery [113,114,115]. In the case of failed back surgery, PEN can be an alternative, given poor reoperation or transforaminal nerve root injection outcomes [38,116].

A modified form of PEN involves balloon adhesiolysis [117,118]. This procedure is known to reduce nerve compression through the insertion of a long, thin catheter with a built-in balloon into the spine through the sacral hiatus, then inflating the balloon to secure space for the lumbar nerve roots to pass [117,118].

The studies investigating the effects of PEN are described in Table 3 [113,114,116,119,120,121].

#### 6.2.4. Radiofrequency Nucleoplasty

Radiofrequency nucleoplasty can be used as an alternative to surgical treatment for discogenic lower back pain [122,123]. This treatment can also be used for the treatment of radiating pain caused by cervical disc herniation [124,125]. While RF neurotomy involves neural ablation, RF nucleoplasty removes the disc matrix [122,123,124,125].

RF nucleoplasty can assist in annulus fibrosus recovery while severing the nociceptive nerves that have grown into the cracked annulus fibrosus [122,123,124,125]. In addition, a portion of the nucleus pulposus can be incinerated with a high frequency, or physically removed, to reduce the pressure of the intervertebral disc in attempting to reposition the protruding intervertebral disc. As such, spinal interventions to fully remove lesions and minimally invasive treatments are becoming similar.

The studies related to radiofrequency nucleoplasty are described in Table 4 [122,126].

## 7. Changes in How Pain Is Viewed

Previously, understanding pain involved using the end-organ dysfunction model, in which structural abnormalities at the pain-causing site were deemed to be the root cause [127]. In terms of this model, pain is relieved through correcting or removing the structural abnormality that caused pain owing to tissue damage or inflammation. However, several cases of pain or certain types of diseases cannot be treated or understood in terms of this model in actual clinical settings. For example, despite the absence of structural abnormalities in many tests, some patients complain of severe chronic pain in fibromyalgia, which is challenging to treat. With the recent introduction of the altered nervous system processing model, which views pain as a pain signal processing problem in the altered nervous system, various pain phenomena can be better understood. Problems encoding or processing sensory information correspond to this model, and physiological changes, genetic predisposition, and psychiatric variables are used for interpretation in this model [127]. Functional MRI for patients with back pain sometimes shows reduced brain volume or areas of activity that are not observed in healthy individuals, and such findings have changed the approach to pain that viewed it as being a structural spinal issue [128,129,130]. This new way of considering pain has deepened the understanding of many patients whose pain has not been controlled by surgery or injection treatment. It is necessary to take a comprehensive therapeutic approach through applying different treatments such as psychological therapy, various pharmacological therapies, and exercise therapy, rather than limiting treatment to surgery or injection therapy when treating pain in these patients [131].

## 8. Patients Requiring Surgery

Degenerative spinal disease results in functional challenges for patients. These challenges do not require strict clinical decisions to be made, as in oncology or cardiovascular disease, where decision making directly affects patients’ lives. Malignant tumors or myocardial infarctions involve serious risks to life if standard protocols and treatments are not followed, whereas degenerative spinal diseases involve inconveniences and are not life-threatening; therefore, modifying treatment decisions flexibly according to each patient’s situation is possible. There is no known therapeutic window or case in which surgery is mandatory but only cases where surgery is recommended. If surgery was safe and ensured an excellent regenerative ability to restore spinal function, it would be recommended even when symptoms were not severe; however, surgery is an invasive treatment that only removes what is present or fixes in place what is out of place [8]. Therefore, it is appropriate to provide surgical treatment only when absolutely necessary.

When considering whether surgery is indicated, while there are no absolute criteria, abnormal neurological findings provide the most critical indications for surgery, followed by imaging findings, nerve conduction test results, and patient preferences. Therefore, the following criteria are recommended for assessing whether patients require surgery [132,133,134]: (i) a high degree of disc herniation resulting in cauda equina syndrome; (ii) a severe degree of lower limb paralysis; (iii) persistent radiating pain that has been unresponsive to conservative treatment for an extended period of time (usually >6 months); and (iv) recurrent and radiating pain sufficient to interfere with activities of daily living. The first two criteria are objective, whereas the latter two are subjective criteria. There is no consensus among clinicians concerning the definition of an appropriate period of conservative treatment, and interference with activities of daily living may vary among individual patients. Therefore, surgery may or may not be necessary depending on a patient’s perceived symptom severity, even when test results are similar.

Failed back surgery syndrome and post-lumbar-surgery syndrome are both potential postoperative complications of spinal surgery [135]. Some patients have residual pain despite successful surgery. Some studies have reported incidence rates as high as 40% for these syndromes. Taken together, good indications for surgery, i.e., cases likely to have a high success rate for spinal surgery, could include HLD with dominant leg pain, spinal stenosis presenting with typical neurogenic intermittent claudication signs and symptoms, mechanical back pain, or spondylolisthesis accompanied with stenosis.

## 9. Conclusions

If there are no significant neurological symptoms, spinal injection should be considered as the initial option rather than surgery. Rather than claiming that spinal injection is more effective than surgery, it should be considered as a prior option when taking into account the failure rates, complications, and treatment costs associated with spinal surgery. Diagnostic injections are employed to identify the source of pain before a patient undergoes therapeutic injection or surgery. Spinal injections help alleviate pain, enhance the quality of life, and could potentially avoid certain surgical procedures. In addition, applying various other conservative treatments in addition to spinal injections is likely to help improve the treatment effect or treatment success rates in such patients.

## Figures and Tables

**Table 1:** Summary of randomized controlled trials for evaluating the efficacy of transforaminal nerve root injections.

Study	Study Design	Participants	Interventions	Outcome Measurement	Summary of Outcomes
Devulder et al., 1999 [89]	Randomized controlled trial	*n* = 60 (three treatment groups with 20 participants each)	Group I with 1 mL bupivacaine 0.5% combined with 1500 units hyaluronidase and 1 mL saline per nerve root sleeve. Group II with 1 mL bupivacaine 0.5% combined with 40 mg methylprednisolone solution per nerve root. Group III with bupivacaine 0.5% combined with 1500 units hyaluronidase and 40 mg methylprednisolone solution.	Verbal pain rating scale at 1, 3, and 6 months	Three treatment methods provided pain relief at the 1-month follow-up, but these effects diminished during the 3- and 6-month follow-ups. Ultimately, none of the three injected solutions demonstrated satisfactory outcome in terms of pain relief.
Karppinen et al., 2001 [94]	Randomized controlled trial	*n* = 160 (two treatment groups with 80 participants each)	Group I: periradicular infiltration with Methylprednisolone-Bupivacaine. Group II: periradicular infiltration with saline.	VAS and Nottingham health profile at 2 weeks, and 1, 3, 6, and 12 months	At the 2-week follow-up, the steroid injection exhibited superior recovery in terms of leg pain, straight leg raising, lumbar flexion, and patient satisfaction. However, the saline infiltration was significantly lower in back pain at 3 and 6 months, as well as lower in leg pain at 6 months. The combination of methylprednisolone and bupivacaine appeared to have a positive short-term effect. However, at 3 and 6 months, the steroid injection showed a “rebound” phenomenon.
Bonetti et al., 2005 [85]	Randomized controlled trial	*n* = 306 (80 in group I, 86 in group II, 70 group III, 70 in group IV)	Group I, including patients with disc disease: 2 mL steroid injection. Group II, including patients with disc disease: infiltration of 3 mL O(2)-O(3) gas mixture. Group III, including patients without disc disease: 2 mL steroid injection. Group IV, including patients without disc disease: infiltration of 3 mL O(2)-O(3) gas mixture.	Modified version of the McNab method at 1 week, and 3 and 6 months	Both treatment methods demonstrated excellent pain reduction effects throughout all follow-up periods, regardless of the presence or absence of disc disease, with the most favorable outcomes observed in the short-term follow-up. The O(2)-O(3) gas mixture provided significantly greater pain relief compared to steroid injections, making it a potential first-line alternative to epidural steroids.
Ackerman et al., 2007 [84]	Randomized controlled trial	*n* = 90 (three treatment groups with 30 participants each)	Group I: lumbar epidural steroid injection using caudal approach with 3 mL of isohexol 300 and 4 mL of preservative-free saline with 40 mg of triamincolone. Group II: lumbar epidural steroid injection using interlaminar approach with 3 mL of isohexol 300 and 19 mL of preservative-free saline with 40 mg of triamcinolone. Group III: lumbar epidural steroid injection using transforaminal approach with 3 mL of isohexol 300 and 40 mg of triamcinolone in 4 mL of preservative-free saline.	VAS at 12 and 24 weeks	During the evaluation period, a significantly higher number of patients who underwent the transforaminal approach reported overall or partial pain relief. The transforaminal route for epidural steroid placement was found to be more effective than the caudal or interlaminar routes.
Jeong et al., 2007 [92]	Randomized controlled trial	*n* = 239 (112 in group I, 127 in group II)	Group I with transforaminal epidural steroid injection using a preganglionic approach. Group II with transforaminal epidural steroid injection using a ganglionic approach.	VAS at 1 and 6 months	In the short-term follow-up, the preganglionic group exhibited superior treatment outcomes compared to the ganglionic group. No significant difference was identified at the medium-term follow-up. These findings suggest that utilizing transforaminal epidural steroid injection with a preganglionic approach is more effective than a ganglionic approach in the short term, and it demonstrates comparable effectiveness to the ganglionic approach in the medium term.
Tafazal et al., 2009 [100]	Randomized controlled trial	*n* = 150 (76 in group I, 74 in group II)	Group I: local anesthetic injection with 2 mL of 0.25% bupivacaine, Group II: peri-radicular infiltration of corticosteroids with 2 mL of 0.25% bupivacaine and 40 mg of methylprednisolone.	VAS and ODI at 6 and 12 weeks, and 12 months	After a 3-month follow-up, there were no statistically significant distinctions in pain relief and functional improvement between the two treatment approaches. Similarly, at a minimum of 1 year following the injection, no variation was observed in the necessity for subsequent interventions between the two methods. The peri-radicular infiltration of corticosteroids for sciatica does not confer any additional advantages when compared to the administration of local anesthetic injection alone.
Ghahreman et al., 2010 [90]	Randomized controlled trial	*n* = 150 (28 in group I, 27 in group II, 37 in group III, 28 in group IV, 30 in group V)	Group I: transforaminal steroid injection with 0.75 mL of 0.5% bupivacaine followed by 1.75 mL of triamcinolone in a concentration of 40 mg/mL. Group II: transforaminal injection of local anesthetic with 2 mL of 0.5% bupivacaine. Group III: transforaminal injection of 2 mL normal saline. Group IV: intramuscular steroid injection with 1.75 mL of triamcinolone (40 mg/mL). Group V: intramuscular normal saline injection with 1.75 mL of triamcinolone (40 mg/mL).	NRS at 3, 6, and 12 months	A notable increase in the number of patients experiencing pain relief was observed with transforaminal injection of steroids compared to those who received transforaminal injection of local anesthetic or saline, intramuscular steroids, or intramuscular saline. However, it is important to note that the proportion of patients with sustained pain relief decreases over time, and only a few patients maintain relief beyond 12 months. The transforaminal injection of steroids is considered to be effective for pain relief in a subset of patients.
Rados et al., 2011 [99]	Randomized controlled trial	*n* = 64 (32 in group I, 32 in group II)	Group I with transforaminal epidural steroid injection of 40 mg methylprednisolone, 3 mL of 0.5% lidocaine. Group II with interlaminar epidural steroid injection of 80 mg of methylprednisolone mixed with 8 mL of 0.5% lidocaine.	VAS and ODI at 3 and 6 months	During the 6-month follow-up period, the outcomes of pain relief and functional improvement were positive for both transforaminal and interlaminar epidural steroid injections. When using the transforaminal approach, it provided slightly better long-term pain relief and functional improvement. However, there was no statistically significant difference between the two treatment methods.
Cohen et al., 2012 [86]	Randomized controlled trial	*n* = 84 (30 in group I, 28 in group II, 26 in group III)	Group I with saline. Group II with corticosteroid. Group III with etanercept.	NRS and ODI at 1, 3, and 6 months	After one month of treatment, overall positive effects were reported, and epidural steroid therapy showed greater efficacy in functional improvement and pain reduction compared to saline or etanercept treatment. Epidural steroid injections have the advantage of providing short-term pain relief for patients with lumbosacral radiculopathy.
Ghai et al., 2014 [91]	Randomized controlled trial	*n* = 62 (32 in group I, 30 in group II)	Group I with fluoroscopically guided epidural injection of methylprednisolone (80 mg) through parasagittal interlaminar approach. Group II with fluoroscopically guided epidural injection of methylprednisolone (80 mg) through transforaminal approach.	VAS and ODI at 2 weeks, and 1, 2, 3, 6, 9, and 12 months	Significant pain relief and function improvement were observed at all time points post-intervention compared to baseline in both groups. The parasagittal interlaminar and transforaminal approach for low back pain yield similar pain relief and functional improvement. The parasagittal interlaminar approach can be considered as a suitable alternative, for equivalent efficiency, better safety profile, and technical ease, to the transforaminal approach.
Kennedy et al., 2014 [95]	Randomized controlled trial	*n* = 78 (41 in group I, 37 in group II)	Group I with dexamethasone. Group II with triamcinolone.	NRS and ODI at 2 weeks, and 3 and 6 months	Both triamcinolone and dexamethasone demonstrated significant improvements in pain and function at 2 weeks, 3 months, and 6 months, with no distinct disparities between the two treatments. Dexamethasone seems to be equally effective as triamcinolone in managing the condition.
Manchikanti et al., 2014 [97]	Randomized controlled trial	*n* = 120 (two treatment groups with 60 participants each)	Group I with 1.5 mL of 1% preservative-free lidocaine, followed by 0.5 mL of sodium chloride solution. Group II with 1% lidocaine, followed by 3 mg, or 0.5 mL of betamethasone.	NRS and ODI at 3, 6, 12, 18, and 24 months	The two-year follow-up results of local anesthesia alone or in combination with steroid therapy are positive. Both local anesthesia with or without steroids in epidural injections can be effective treatments for patients with disc herniation or radiculopathy. These findings indicate that the superiority of steroids over local anesthesia is insufficient in the two-year follow-up survey.
Denis et al., 2015 [88]	Randomized controlled trial	*n* = 56 (29 in group I, 27 in group II)	Group I with lumbar transforaminal injection of dexamethasone 7.5 mg, Group II with lumbar transforaminal injection of betamethasone 6.0 mg,	VAS and ODI at 1, 3, and 6 months	At 3 months, there was no significant difference between the two treatments in terms of pain relief and functional improvement. However, at 6 months, the dexamethasone treatment showed better effects in terms of functional improvement.
Kamble et al., 2016 [93]	Randomized controlled trial	*n* = 90 (three treatment groups with 30 participants each)	Group I with transforaminal steroid injection. Group II with caudal steroid injection. Group III with epidural steroid.	VAS and ODI at 1, 6, and 12 months	The transforaminal route showed greater improvements in pain relief and functional improvement compared to the interlaminar and caudal routes. However, there was no significant difference between the interlaminar and caudal routes. Overall, the transforaminal steroid injection group demonstrated better symptomatic improvement in both the short and long term compared to the interlaminar and caudal steroid injection groups.
Pandey, 2016 [98]	Randomized controlled trial	*n* = 140 (82 in group I, 40 in group II, 18 in group III)	Group I with injection by caudal route. Group II with injection by transforaminal route. Group III with injection by interlaminar route.	JOA at 6 and 12 months	After 12 months of administering steroid injections, all three routes showed effectiveness in improving the JOA score. However, the transforaminal route was significantly more effective than the caudal and interlaminar routes at both 6 and 12 months after the injection. There was no significant difference observed between the caudal and interlaminar routes in terms of their effectiveness.
Makkar et al., 2019 [96]	Randomized controlled trial	*n* = 61 (21 in group I, 20 in group II, 20 in group III)	Group I with epidural steroid injection using midline interlaminar approach. Group II with epidural steroid injection using parasagittal interlaminar approach. Group III with epidural steroid injection using transforaminal approach.	VAS and ODI at 2 and 4 weeks, and 3 and 6 months	The parasagittal interlaminar approach and transforaminal approach had significantly higher rates of effective pain relief compared to the midline interlaminar approach at 3 and 6 months. ODI scores were significantly lower in the parasagittal interlaminar approach and transforaminal approach compared to the midline interlaminar approach, but there was no significant difference between parasagittal interlaminar approach and transforaminal approach.
De et al., 2020 [87]	Randomized controlled trial	*n* = 50 (two treatment groups with 25 participants each)	Group I with transforaminal epidural local anesthetic injection of 1 mL 0.5% bupivacaine. Group II with transforaminal epidural injection of 1 mL 0.5% bupivacaine with 3 cycles of pulsed radiofrequency of the dorsal root ganglion for 180 s.	VAS and ODI at 2 weeks and 1, 2, 3, and 6 months	The lumbar pulsed radiofrequency group showed statistically significant reductions in both pain and functional improvement compared to the transforaminal epidural local anesthetic injection group from 2 weeks to 6 months. The application of pulsed radiofrequency to the DRG for an extended period provides long-term pain relief and improves the functional quality of life in patients with chronic lower back pain.
Wei et al., 2020 [101]	Randomized controlled trial	*n* = 90 (three treatment groups with 30 participants each)	Group I with TNF-α inhibitor. Group II with steroids. Group III with lidocaine-only.	VAS and modified ODI at 6 months	The TNF-α inhibitor showed significantly greater pain relief and improvement in movement function compared to steroids and lidocaine. There was no significant difference between the effects of steroids and lidocaine.

VAS, visual analog scale; ODI, Oswestry disability index; JOA, Japanese Orthopaedic Association scale; NRS, numeric rating scale.

**Table 2:** Summary of randomized controlled trials for evaluating the efficacy of therapeutic medial branch blocks.

Study	Study Design	Participants	Interventions	Outcome Measurement	Summary of Outcomes
Manchikanti et al. 2001 [110]	Randomized controlled trial	*n* = 73 (32 in group I and 41 in group II)	Group I with local anesthetic and Sarapin. Group II with local anesthetic, Sarapin, and methyl prednisolone.	Pain relief, physical health, psychological status, narcotic intake, and employment status at 1, 3, 6, 12,18, 24, and 32 months.	Relief from one to three injections decreased with time, with the highest relief in the first 3 months. The treatment showed significant improvements in overall health status, including pain relief, physical, functional, and psychological status, as well as the ability to return to work. In conclusion, medial branch blocks with local anesthetic and Sarapin, with or without steroids, proved to be a cost-effective and beneficial treatment option for improving pain status, physical and psychological well-being, functional status, and the ability to return to work.
Manchikanti et al. 2007 [111]	Randomized controlled trial	n = 60 (four treatment groups with 15 participants each)	Group I with bupivacaine only. Group II with bupivacaine and Sarapin. Group III with bupivacaine and steroids. Group IV with bupivacaine, Sarapin, and steroids.	NRS, ODI, opioid intake, and employment status at 3, 6, and 12 months.	Significant pain relief and functional improvement were observed at 3, 6, and 12 months. No significant difference was found between steroid and non-steroid treatment groups. The treatment of lumbar facet joint nerve block using local anesthesia, with or without Sarapin or steroids, may be effective for chronic lower back pain originating from the facet joint.

NRS: numeric rating scale, ODI: Oswestry disability index.

**Table 3:** Summary of studies for evaluating the efficacy of percutaneous epidural neuroplasty.

Study	Study Design	Participants	Interventions	Outcome Measurement	Summary of Outcomes
Gerdesmeyer et al., 2013 [120]	Randomized controlled trial	*n* = 90 (44 in group I and 46 in group II)	Group I: The caudal approach involved the intentional insertion of a needle and catheter without penetrating the spinal canal. The catheter was placed into the subcutaneous tissue above the affected area, and for three days, 10 mL of preservative-free sodium chloride solution was administered through the catheter before its removal. Group II: The Tun-L catheter was positioned through the sacral canal with injection of 10 mL of contrast. Local anesthetic, 10 mL, 0.25% bupivacaine was administered through the catheter, followed by 10 mL of preservative-free sodium chloride solution infused with 150 units per mL of hyaluronidase. Slow injections of sodium chloride solution, 10 mL, 10%, containing 40 mg of triamcinolone, along with 2 mL of 0.25% bupivacaine. On the 2nd and 3rd days, 10 mL of 0.25% bupivacaine was injected through the catheter, followed by slow injection of 10 mL of 10% sodium chloride solution and 2 mL, 0.25% bupivacaine.	VAS and ODI at 3, 6, and 12 months	In the lysis group, pain relief and functional improvement were significantly greater at 3, 6, and 12 months compared to the placebo group. The minimally invasive percutaneous adhesiolysis procedure could be considered as the first treatment option for patients with chronic lumbosacral radicular pain.
Ji et al., 2015 [116]	Retrospective	*n* = 363	Catheterization was performed with a caudal approach. After final positioning of the Racz catheter, 6 mL of 0.2% preservative free ropivacaine containing 1500 units of hyaluronidase and 4 mL of 40% triamcinolone acetate was injected. An hour later, 6 mL of 8% sodium chloride solution was infused over 30 min in the recovery room while being monitored.	VAS and Odom’s criteria at 3, 6, 12, and 24 months	Percutaneous epidural neuroplasty proves to be an effective intervention for managing lumbar disc herniation at a single level without affecting the dural sac cross-sectional area.
Moon et al., 2017 [121]	Retrospective	*n* = 407	The percutaneous epidural adhesiolysis was performed using an RK needle and Racz catheter through the caudal approach. Following the accurate placement of the catheter in the anterior epidural space at the target site, a test dose of 3–5 mL of 1% lidocaine was administered. Subsequently, 10 mL of 0.9% sodium chloride solution was injected, followed by a mixture of 0.125% bupivacaine and 5 mg of dexamethasone. After 5 min, under real-time fluoroscopic guidance, 10 mL of 10% sodium chloride solution was slowly injected.	NRS and GPES at 1 and 12 months	After 12 months of percutaneous epidural adhesiolysis, a highly effective pain reduction was observed in 72.2% of patients. Those who experienced pain relief reported a high level of treatment satisfaction.
Cho et al., 2019 [113]	Retrospective	*n* = 430	The treatment involved percutaneous adhesiolysis or neuroplasty through a caudal approach, with catheter placement ventrally and laterally. After ensuring proper catheter positioning, and contrast injection, a mixture of 6 mL of 0.2% preservative-free ropivacaine with 1500 units of hyaluronidase and 4 mL of betamethasone sodium phosphate was injected. An hour later, 6 mL of 8% sodium chloride solution was infused over 30 min in the recovery room while being monitored, and finally, the epidural catheter was removed.	VAS and Odom’s criteria at 1, 3, 6, and 12 months	The back and leg pain significantly decreased during the entire follow-up period after percutaneous epidural neuroplasty. Both short-term and long-term outcomes were positive. These results indicate that percutaneous epidural neuroplasty is an effective treatment for pain in the back and legs caused by a single-level lumbar disc herniation, and the outcomes are not influenced by the type of lumbar disc herniation.
Park et al., 2018 [114]	Retrospective	*n* = 78	The procedure was performed using a caudal entry approach. After placing the catheter in the appropriate position, a mixture of 10 mL of 0.9% sodium chloride solution and 300 units of hyaluronidase was injected. Another epidurogram was performed, followed by the slow injection of 8 mL of 0.2% ropivacaine and 40 mg of triamcinolone.	VAS, ODI, and SF-12 at 1, 3, 6, and 12 months	During the follow-up period, there was a notable improvement in back and leg pain relief, functional improvement, and overall health. It is possible that extraforaminal contrast distribution during lumbar percutaneous epidural neuroplasty could be linked to enhanced functional outcomes.
Choi et al., 2017 [119]	Retrospective	*n* = 543 (333 in group I and 210 in group II)	Group I: 5% hypertonic sodium chloride solution injection. Group II: 10% hypertonic sodium chloride solution injection. The caudal approach was used for the procedure, and catheterization was performed after reaching the final catheter position. A total of 5 mL of 0.25% ropivacaine, along with 1500 units of hyaluronidase was injected. Following the confirmation of no complications, 6 mL of 10% or 5% sodium chloride solution was injected, at a rate of 1 mL every 15 min. Subsequently, 2 mL of 0.9% sodium chloride solution containing 40 mg of triamcinolone was injected.	NRS at 1, 3, and 6 months	Both 5% and 10% hypertonic sodium chloride solution injections significantly reduced pain at 1, 3, and 6 months after percutaneous epidural adhesiolysis when compared to pre-procedure levels. However, no significant differences were observed between the 5% and 10% groups during the follow-up period at any point. For patients with concerns about cytotoxic side effects or infusion-related pain, the use of 5% hypertonic saline could be considered as an alternative to the 10% hypertonic saline.

VAS, visual analog scale; ODI, Oswestry disability index; NRS, numeric rating scale; GPES, global perceived effect scale; SF-12, 12-item short-form health survey.

**Table 4:** Summary of studies for evaluating the efficacy of radiofrequency nucleoplasty.

Study	Study Design	Participants	Interventions	Outcome Measurement	Summary of Outcomes
Adakli et al., 2015 [122]	Retrospective	*n* = 73 (36 in group I and 37 in group II)	Group I with lumbar radiofrequency thermocoagulation nucleoplasty. Group II with targeted disc decompression.	VAS and FRI at 1, 6, and 12 months	A significant improvement was observed in all pain relief and improvement of function when compared with the preprocedure values, in both methods. The degree of pain relief after 1, 6 and 12 months was significantly lower in the decompression compared to nucleoplasty, but there was no statistically significant difference in function improvement. These results indicate that radiofrequency thermocoagulation nucleoplasty and targeted disc decompression could be effective and safe alternatives to surgery for the treatment of hernia nucleus pulposus.
Nie et al., 2018 [126]	Retrospective	*n* = 260 (113 in group I and 147 in group II)	Group I with nucleoplasty. Group II with targeted disc decompression.	VAS and FRI at 1, 3, 6, 12, 24, and 60 months	The findings from a 5-year follow-up study revealed that both targeted disc decompression and nucleoplasty effectively reduced pain caused by disc herniation and improved the patients’ quality of life. Furthermore, there was no statistically significant difference in therapeutic efficacy between these two treatment methods.

VAS, visual analog scale; FRI, functional rating index.

## Data Availability

No new data were created or analyzed in this study. Data sharing is not applicable to this article.
